# Apparent Recovery from Phthesis

**Published:** 1887-01

**Authors:** 


### Apparent Recovery From Phthesis.

A patient sent to the Polyclinic in the fall of 1883, supposed to be dying from consumption, is still alive and apparently healthy, having recovered under treatment by inhalations of compressed air, the diet consisted of raw and rare beef, milk, eggs, and green vegetables. The medicines given from time to time, according to indications, were fluid extract of erythroxylon (coca), wine of coca, pill of iodoform and iron, syrup of phosphites with iron, and compound syrup of phosphate of iron.

THE INFLUENCE OF CLIMATE AND SOIL UPON MEDICINAL PLANTS.—Professor Vogel says that plants do not always contain their characteristic alkaloids when grown under other than natural conditions. Hemlock does not yield coniine in Scotland, and cinchona plants are nearly free from quinine when grown in hothouses. Tannin is found in the greatest quantity in trees which have had a full supply of direct sunlight.

IT is proposed to form an association of the graduates of the Lawrance scientific school connected with Harvard university. There are numbered among these graduates many of the students of Agassiz.

